# Separability of active semantic and phonological maintenance in verbal working memory

**DOI:** 10.1371/journal.pone.0193808

**Published:** 2018-03-07

**Authors:** Ryoji Nishiyama

**Affiliations:** Graduate School of Education, Kyoto University, Kyoto, Japan; Baycrest Health Sciences, CANADA

## Abstract

Models of verbal working memory that incorporate active memory maintenance, long-term memory networks, and attention control have been developed. Current studies suggest that semantic representations of words, evoked via long-term memory networks, are actively maintained until they are needed to fulfill a role. In other words, it is possible that some mechanism actively refreshes semantic representations of words, analogous to but independently from articulatory rehearsal which refreshes phonological representations. One valuable piece of evidence is a double dissociation, observed in a dual task paradigm in which manual tapping disrupted a semantic memory task while articulatory suppression disrupted a phonological memory task. However, in that study, the secondary tasks could have competed not only with the maintenance but also with the encoding activities. Additionally, the study items in the phonological memory tasks were words; hence, the discriminability of the memory tasks is doubtful. The present study, therefore, examined a potential double dissociation in situations where the secondary tasks could not compete with encoding, using a modified phonological memory task. Furthermore, this article discusses a potential mechanism for maintaining semantic representations.

## Introduction

“WASURENEBAKOSO OMOIDASAZUSOUROU” [1]. This passage is quoted from a letter in the Edo period of Japanese history. It translates as, “I will never recall you, because I will never forget you.” This implies that trying to maintain certain representations in mind actively is conceptually distinct from possessing an inactive long term-memory (LTM) structure which is the basis of those representations. Models of working memory (WM) have been developed to describe active maintenance aspects and network aspects of memory (see, [2, 3]). Words evoke semantic representations based on LTM (e.g., [4]). Current evidence suggests that such semantic representations can contribute to performance of short-term memory tasks separably from phonological representations (e.g., [5, 6–10]). If so, are the semantic representations actively maintained independently from phonological maintenance?

*Dissociation from phonological maintenance*. Active maintenance of words has been a focal interest in studies of verbal WM (e.g., [11]). Such studies have developed sophisticated models describing how articulatory rehearsal can refresh decaying phonological representations (e.g., Baddeley’s ‘phonological loop’ model:[12, 13]). Articulatory activity is highly automatized and can be performed without generating attentional demands [14]. Of note here is that semantic representations seem to be preserved even when the articulatory activities cannot adequately function. For instance, articulatory suppression (AS), irrelevant articulations uttered concurrently with memory maintenance, prevents articulatory rehearsal and hence abolishes phonological effects in verbal temporary memory tasks (e.g., [15, 16]); however, semantic effects are preserved under AS [17–19]. Similarly, certain individuals with deficits in articulatory rehearsal demonstrated preservation of semantic effects [20–23]. Such single dissociation is evidence that articulatory rehearsal is dispensable for the preservation of semantic representations. Nevertheless, it is possible that semantic representations can be lasting without maintenance activities and can mediate reconstruction of decaying phonological representations of studied words [24].

However, Nishiyama [25] demonstrated a double dissociation in which tapping disrupted the performance of a modified synonym recognition task [26, 27] and AS disrupted the performance of the homophone recognition task, selectively. The synonym recognition task required participants to choose synonyms for studied words as recognition targets. Hence, the semantic representations were usable but reconstruction of the phonological representations of the study words was not needed. In contrast, the homophone recognition task required participants to choose homophones as recognition targets. In that case, the semantic representations were not needed but the phonological representations were needed. Tapping demands some attention control but does not compete with articulatory rehearsal [28, 29], whereas AS strongly prevents articulatory rehearsal but demands less attention control [28, 30]. In other words, the negligible disruption of the synonym recognition performance by AS confirms that articulatory rehearsal is dispensable for maintaining semantic representations. Rather, the disruption of the synonym recognition performance by tapping suggests that some attention-demanding activity is involved in maintaining semantic representations of words, analogous to the articulatory rehearsal that maintains phonological representations. This is consistent with another finding involving a double dissociation in lexical decision speeds, performed within synonym and rhyme recognition tasks [31]. Moreover, such double dissociation is consistent with some models of WM. These suppose that two maintenance mechanisms, i.e., articulatory rehearsal and attentional refreshing, are involved in the active maintenance of words [32–35]. Of note, these models assume that attentional refreshing can handle semantic representations.

Nevertheless, there are several concerns regarding that study. One stems from the use of words in the homophone recognition task because semantic representations can be evoked at encoding. One study suggested that semantic representations could support serial recall performance without being interfered with by a concurrent attention-demanding task [36]. Hence, the null interference by tapping on homophone recognition performance might be attributed to semantic representations supporting phonological retrieval. This potential contamination of the measurement task may weaken the claim that the double dissociation reflects a difference in phonological and semantic maintenance mechanisms. Another, more severe, concern stems from performing the secondary tasks throughout the study phase. Such secondary tasks could compete not only with memory maintenance but also with encoding. Specifically, articulatory activities are crucial for phonological encoding via printed words [12], so the disruption of the homophone recognition task by AS could occur at encoding. On the other hand, it is unclear whether attentional demand can influence semantic encoding [37, 38] or not [39]. In any case, the double dissociation is not definitive evidence for the separability of the maintenance mechanisms.

Present study. The principal purpose of this study is to verify whether the double dissociation [40] reflects a difference in maintenance mechanisms for semantic and phonological representations. Experiments 1A-C attempted to replicate the selective dual task effects on semantic and psychological memory performance, while modifying the phonological memory task. The stimuli used in the modified task were nonwords, hereafter referred to as “nonword recognition”; hence, semantic influence can be eliminated. Experiments 2A and 2B examined the dual task effects in situations where tapping and AS should not compete with encoding. Double dissociation in these situations should confirm that the maintenance mechanisms are different. Addressing this issue would provide a comprehensive understanding of active maintenance mechanisms in verbal working memory.

## General methods

The research was reviewed and approved by the Kwansei Gakuin University Institutional Review Board for Behavioral Research with Human Participant (2015–17). A dual task paradigm, consisting of two memory recognition tasks and two secondary tasks, was used in all experiments. The memory tasks were a synonym recognition task and a nonword recognition task; the secondary tasks were tapping and articulatory suppression (AS).

### Synonym recognition task

Participants were instructed to retain words by imagining their meanings (a mental picture, sound, taste, emotion, or other experiences without articulation) and reimagining them to keep the words in mind without articulating them. Then, they were required to choose synonyms as recognition targets. The stimuli were words consisting of either two kanji characters or a kanji and a hiragana character whose imageability ratings were above 4.0 (7-point scale: [41]). Kanji is a logographic writing system in which characters have different pronunciations based upon context. Articulatory activities are not always needed to access meanings of words composed of Kanji characters [42]. Synonyms were selected based on a Digital Synonym Dictionary (Gengo-Kogaku Kenkyujo inc.). Each study word, its recognition target, and four distractors constituted a fixed set in which the imageability rating differences across the six words did not surpass 1.0. Ten fixed sets constituted a single list, and 12 lists were constructed.

### Nonword recognition task

Participants were instructed to retain words through articulatory rehearsal without thinking about anything else. The stimuli were meaningless letter strings selected from Umemoto, Morikawa and Ibuki [43]. The study words were displayed in Hiragana characters while recognition targets were displayed in Katakana characters. Both sets of characters represent the same phonetic sounds, yet they are not orthographically the same. The study words, the recognition targets and the distractors made up 12 lists in the same fashion as in the synonym recognition task.

### Tapping

Participants were instructed to tap the 0 key on the computer keyboard at a rate of 2 responses per second in Experiments 1A and 2A, and at a rate of 1.5 responses per second in Experiment 2B (see S1 Table). The pace of the secondary tasks, 1–2 responses per 1 second, was more standard than the pace in Nishiyama [2], where 1 response per 2 seconds was used.

### Articulatory suppression

Participants were instructed to articulate “the” at a rate of 2 responses per second in Experiment 1B, at a rate of 1 per second in Experiments 1C and 2A, and at a rate of 1.5 responses per second in Experiment 2B (see S1 Table).

### Procedure

All stimuli were presented on a CRT monitor, using a program written in the Matlab application, powered by an IBM ThinkPad T60 computer. Tapping and AS were recorded by the computer (see S1 Table).

During the study phase, ten words appeared for 1 s per word with a 2 s interstimulus interval (Experiments 1A-C & 2A) or without any interstimulus interval (Experiment 2B). The recognition phase started immediately after the last study word disappeared (Experiments 1A-C & 2A) or after a six-second delay (Experiment 2B). Five words (a target and four distractors) were simultaneously presented in a horizontal arrangement; participants chose a target for each of the study items by pressing the keyboard. Targets appeared in the same order as in the study phase. Half of the participants performed the synonym recognition task first and the other half performed the nonword recognition task first. In each memory task, participants completed two practice trials (single, dual task), then completed six trials in each of two conditions (single, dual task). The order of the conditions and study words were pseudo-randomized across participants.

## Experiment 1A

The experiment examined the effects of tapping (2 taps per second), performed throughout the study phase, on synonym and nonword recognition performance. Tapping demands attention control but does not compete with articulatory rehearsal [28, 29]. If some attention-demanding mechanism other than articulatory rehearsal maintains or encodes semantic representations, selective disruption by tapping of synonym recognition performance would emerge (S1 Table).

### Participants

Twelve Japanese undergraduate and graduate students participated in each experiment (10 females, mean age = 20.58, SD = 1.0). Written informed consent was obtained from a participant after an explanation of the purpose and procedure of this research. All participants were native speakers of Japanese who had not participated in the other experiments (Experiments 1B, 2A, 2B).

### Results

**Fig 1:**
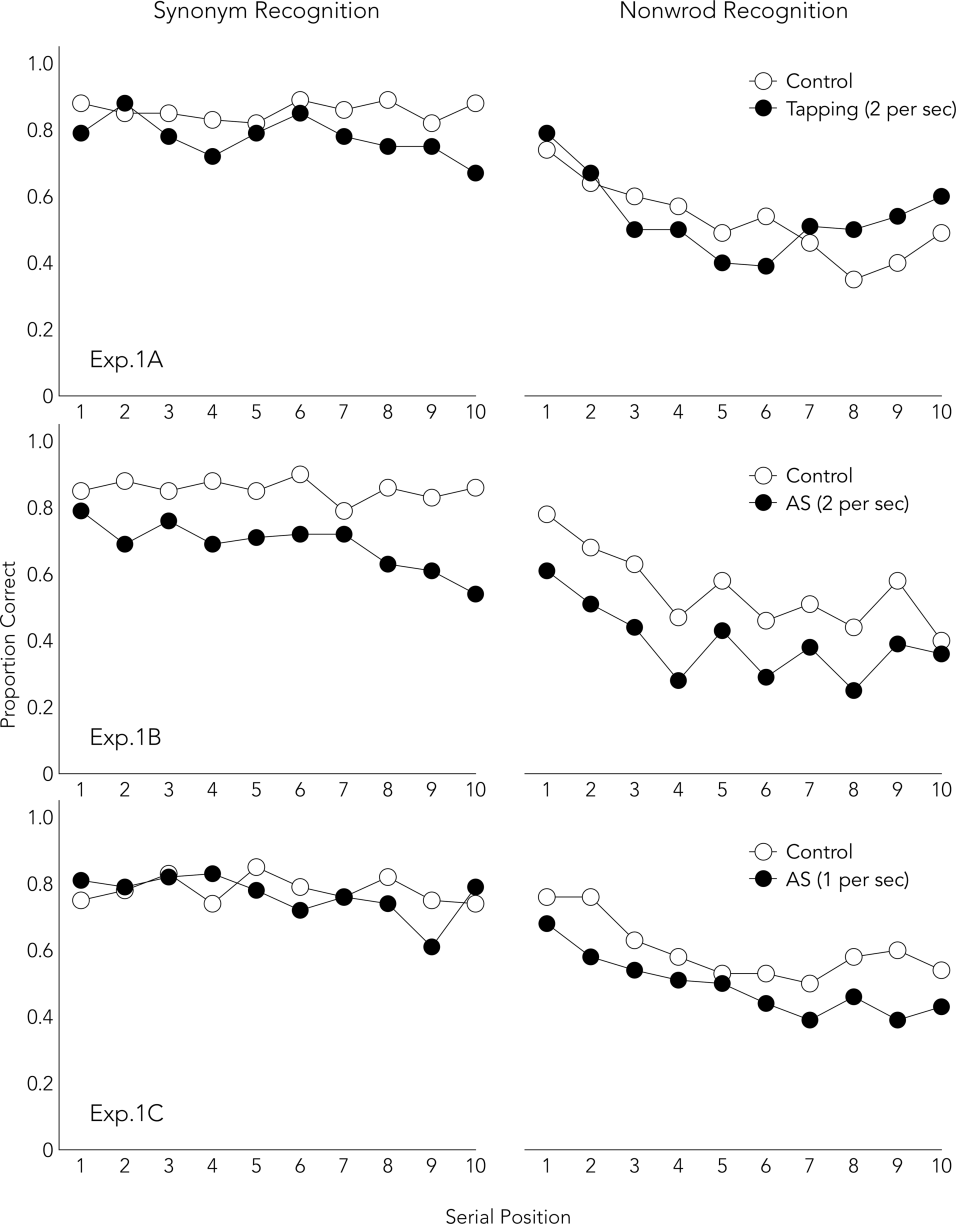
Serial position curves for both conditions in Experiments 1A-1C.

Fig 1 shows the proportion of correct responses in each serial position. Tables 1 and 2 show the mean recognition rates in the memory tasks for each condition as well as the results of a repeated measures ANOVA and a Bayesian ANOVA. Of note, the interaction between memory task and dual task indicates selective disruption of synonym recognition by tapping. The results replicated the findings of Nishiyama [25], using a more appropriate phonological memory task and a secondary task with a more standard pace.

**Table 1:** Mean recognition rate on the memory tasks for each condition, as well as Bayes factor (BF) and effect size for dual task in Experiments 1A, 1B, and 1C.

	Condition					
	Single (*SD*)	Dual (*SD*)	BF	Partial η^2^		
Exp.1A: Tapping (2/sec)						
Synonym	0.86	(0.8)	0.78	(0.11)	196.50	.74
Nonword	0.53	(0.13)	0.54	(0.14)	0.35	.04
Exp.1B: AS (2/sec)						
Synonym	0.85	(0.10)	0.69	(0.18)	25.00	.60
Nonword	0.55	(0.08)	0.39	(0.08)	82.26	.69
Exp.1C: AS (1/sec)						
Synonym	0.78	(0.14)	0.77	(0.13)	0.55	.13
Nonword	0.60	(0.14)	0.49	(0.12)	24.19	.60

*Note*. AS = Articulatory suppression. Decrement was calculated by subtracting single from dual task condition. The data was analyzed through a Bayesian ANOVA (BANOVA: [44]), using the BayesFactor package [45] in R software [46]. Partial η^2^ is based on ANOVAs separately conducted on each secondary task condition of each task (*df* = 1,11). The chance of performing each task correctly was 0.2.

**Table 2:** The results of ANOVAs in Experiments 1A, 1B, and 1C.

	Exp.1A: Tapping (2/sec)			Exp.1B: AS (2/sec)			Exp.1C: AS (1/sec)		
	*F*		η*_p_^2^*	*F*		η*_p_^2^*	*F*		η*_p_^2^*
Memory task	68.38	***	.86	54.11	***	.83	51.53	***	.82
Dual task	4.82		.30	36.03	***	.77	14.61	**	.57
Memory task × Dual task	24.55	***	.69	0.02		< .01	13.24	**	.55

Note.

*** *p* < .001.

** *p* < .01. The *df*s of the main effects and the interaction were 1,11.

## Experiment 1B

This experiment examined effects of AS (2 per second), performed throughout the study phase, on synonym and nonword recognition performance. AS competes more with articulatory rehearsal than with attention control. If articulatory rehearsal is not indispensable for maintaining semantic representations, selective disruption by AS of nonword recognition performance would emerge (S1 Table).

### Participants

Twelve Japanese undergraduate and graduate students participated in each experiment (11 females, mean age = 20.17, SD = 0.83). Written informed consent was obtained from a participant after an explanation of the purpose and procedure of this research. All participants were native speakers of Japanese who had not participated in the other experiments.

### Results

Tables 1 and 2 show that the interaction between the memory task and dual task is not significant due to the disruption of synonym recognition performance by AS. This is not consistent with the results of Nishiyama [25], and suggests that articulatory rehearsal is involved in maintaining semantic representations. However, the rate of AS could have been too fast to be performed automatically; then AS might demand attentional control. Thus, Experiment 1C addressed this by adopting a slower rate of utterance.

## Experiment 1C

This experiment examined the effects of AS on synonym and nonword recognition performance again, modifying the AS rate from 2 responses per second to 1 per second (S1 Table).

### Participants

Twelve Japanese undergraduate and graduate students participated in each experiment (9 females, mean age = 19.75, SD = 1.06). Written informed consent was obtained from a participant after an explanation of the purpose and procedure of this research. All participants were native speakers of Japanese who had not participated in the other experiments.

### Results

Tables 1 and 2 show showed selective disruption of nonword recognition performance by AS. The results replicated the findings of Nishiyama [25], and support the interpretation of the results of Experiment 1B that AS with a fast pace demands attentional control. Overall, the combined results of Experiments 1A-C indicate that some attention-demanding activity is involved in activating (encoding and maintaining) semantic representations. Nevertheless, it remains unclear whether activating semantic representations is required for maintenance or encoding.

## Experiments 2A

The procedures of Experiment 2A were identical to those of Experiments 1A-1C, except that the secondary tasks were performed only during ISIs. The secondary tasks should not compete with the semantic and phonological encoding. Tapping and AS were performed at the rates of two responses per second and one per second, respectively. The type of dual task (tapping, AS) was manipulated as a between-participant variable (S1 Table).

### Participants

Twenty-four Japanese undergraduate and graduate students (13 females, mean age = 20.83, SD = 0.82) participated in the experiments. Written informed consent was obtained from a participant after an explanation of the purpose and procedure of this research. All participants were native speakers of Japanese who had not participated in the other experiments. The participants were divided into two secondary task condition groups.

### Design

Experiment 2A was a 2 (memory task: synonym, nonword recognition) × 2 (dual task: single, dual task) × 2 (secondary task type: tapping, AS) mixed factorial design.

### Results

**Fig 2:**
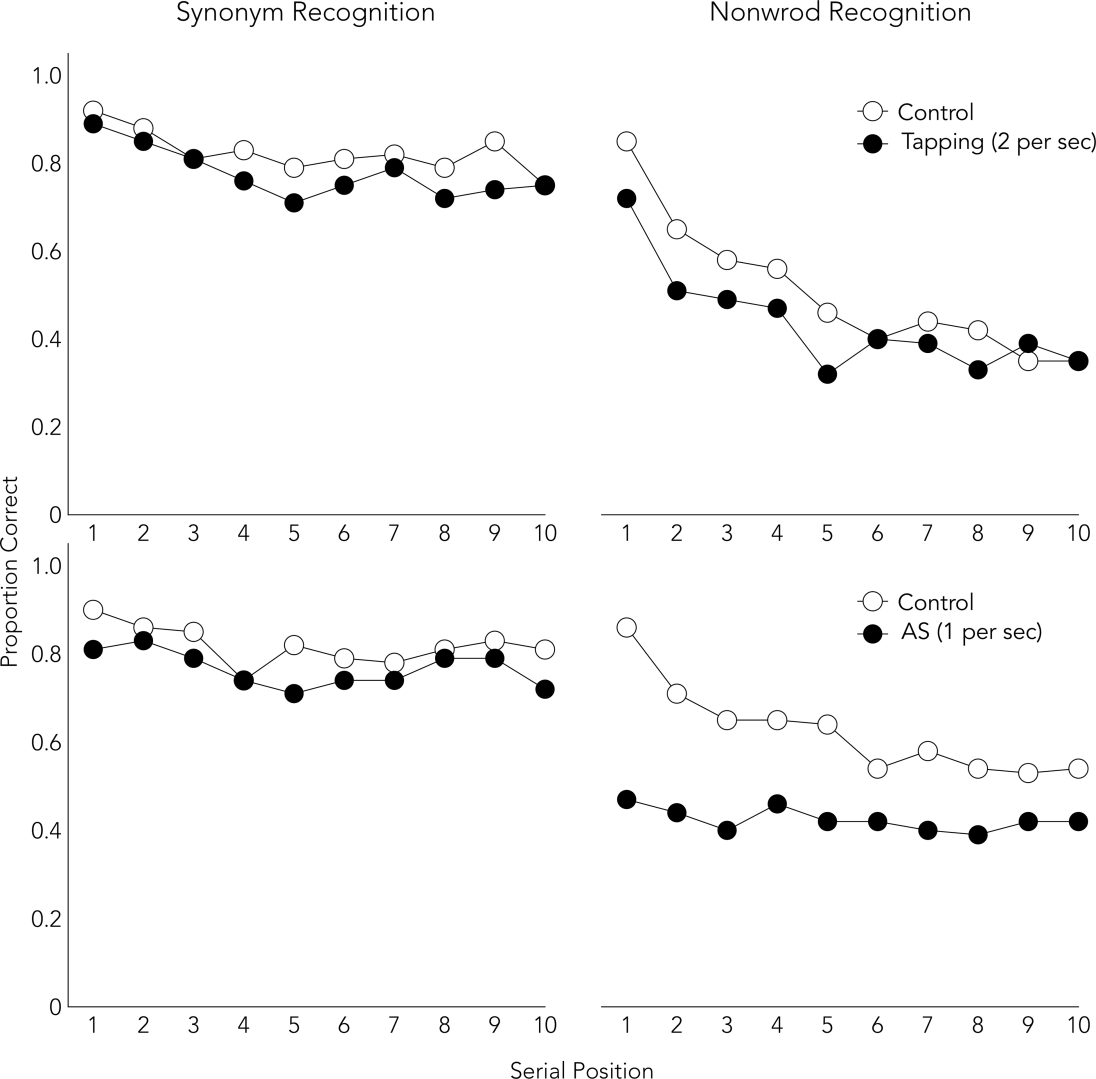
Serial position curves for both conditions in Experiments 2A.

Fig 2 shows the proportion of correct responses in each serial position. Tables 3 and 4 show that the three-way interaction was significant. Nevertheless, both secondary tasks disrupted both types of recognition performance, although AS disrupted nonword recognition performance more severely than synonym recognition performance. This result is not straightforward because the duration of the secondary tasks was shorter in Experiment 2A, where participants performed the secondary tasks only during the ISIs, than in Experiments 1A-1C, where participants performed the secondary tasks throughout the study phase. A possible explanation is that the intervening secondary tasks (performed during ISI) might have impaired the automaticity of articulatory rehearsal. Experiment 2B addressed this issue by using a different procedure for secondary tasks which could compete not with encoding but with maintenance.

**Table 3:** Mean recognition rate on the memory tasks for each condition, as well as Bayes factor (BF) and effect size for dual task in Experiments 2A and 2B.

	Condition					
	Single (*SD*)		Dual (*SD*)		BF	Partial η2
Experiment 2A						
Tapping (2/sec)						
Synonym	0.82	(0.14)	0.78	(0.15)	1.11	.25
Nonword	0.51	(0.13)	0.44	(0.12)	1.73	.31
AS (1/sec)						
Synonym	0.82	(0.12)	0.77	(0.13)	4.75	.44
Nonword	0.63	(0.12)	0.42	(0.16)	5774.41	.87
Experiment 2B						
Tapping (1.5/sec)						
Synonym	0.71	(0.09)	0.60	(0.12)	462.71	.78
Nonword	0.44	(0.08)	0.45	(0.09)	0.30	.01
AS (1.5/sec)						
Synonym	0.67	(0.12)	0.70	(0.11)	0.42	.08
Nonword	0.49	(0.10)	0.36	(0.05)	55.05	.66

*Note*. AS = Articulatory suppression. The decrement was calculated by subtracting single from dual task condition. The data was analyzed through a BANOVA. Partial η^2^ is based on ANOVAs separately conducted on each secondary task condition for each task (*df* = 1,11).

**Table 4:** The results of ANOVAs in Experiments 2A and 2B.

	Exp.2A: ISI			Exp.2B: delay		
	*F*		η*_p_^2^*	*F*		η*_p_^2^*
Secondary task type	0.28		.01	0.08		< .01
Memory task	92.04	***	.81	89.03	***	.80
Dual task	84.11	***	.79	0.91		.04
Secondary task type × Memory task	0.97		.04	15.60	***	.41
Secondary task type × Dual task	11.89	***	.35	0.03		< .01
Memory task × Dual task	8.93	**	.29	0.87		.04
Three-way Interaction	5.08	*	.19	27.57	***	.56

Note.

*** *p* < .001.

** *p* < .01.

* *p* < .05. The *df*s of the main effects and the interaction were 1,22.

## Experiments 2B

This experiment examined the dual task effects when the secondary tasks were performed during a delay interval before the recognition test. The procedure was identical to that of Experiment 2A, except that a six-second delay interval was added before the recognition test, and ISIs were removed (study words were presented for 1 s per word without any ISI). During the delay, in the single task condition participants repeatedly imaged the meanings of words (synonym recognition task) or articulated the words (nonword recognition task). Whereas in the dual task condition participants performed the secondary tasks concurrently with the maintenance activities. Tapping and AS rates were 1.5 responses per second (S1 Table).

### Participants

Twenty-four Japanese undergraduate and graduate students (20 females, mean age = 19.63, SD = 0.77) participated in the experiments. Written informed consent was obtained from a participant after an explanation of the purpose and procedure of this research. All participants were native speakers of Japanese who had not participated in the other experiments. The participants were divided into two secondary task condition groups.

### Design

Experiment 2B was a 2 (memory task: synonym, nonword recognition) × 2 (dual task: single, dual task) × 2 (secondary task type: tapping, AS) mixed factorial design.

### Results

**Fig 3:**
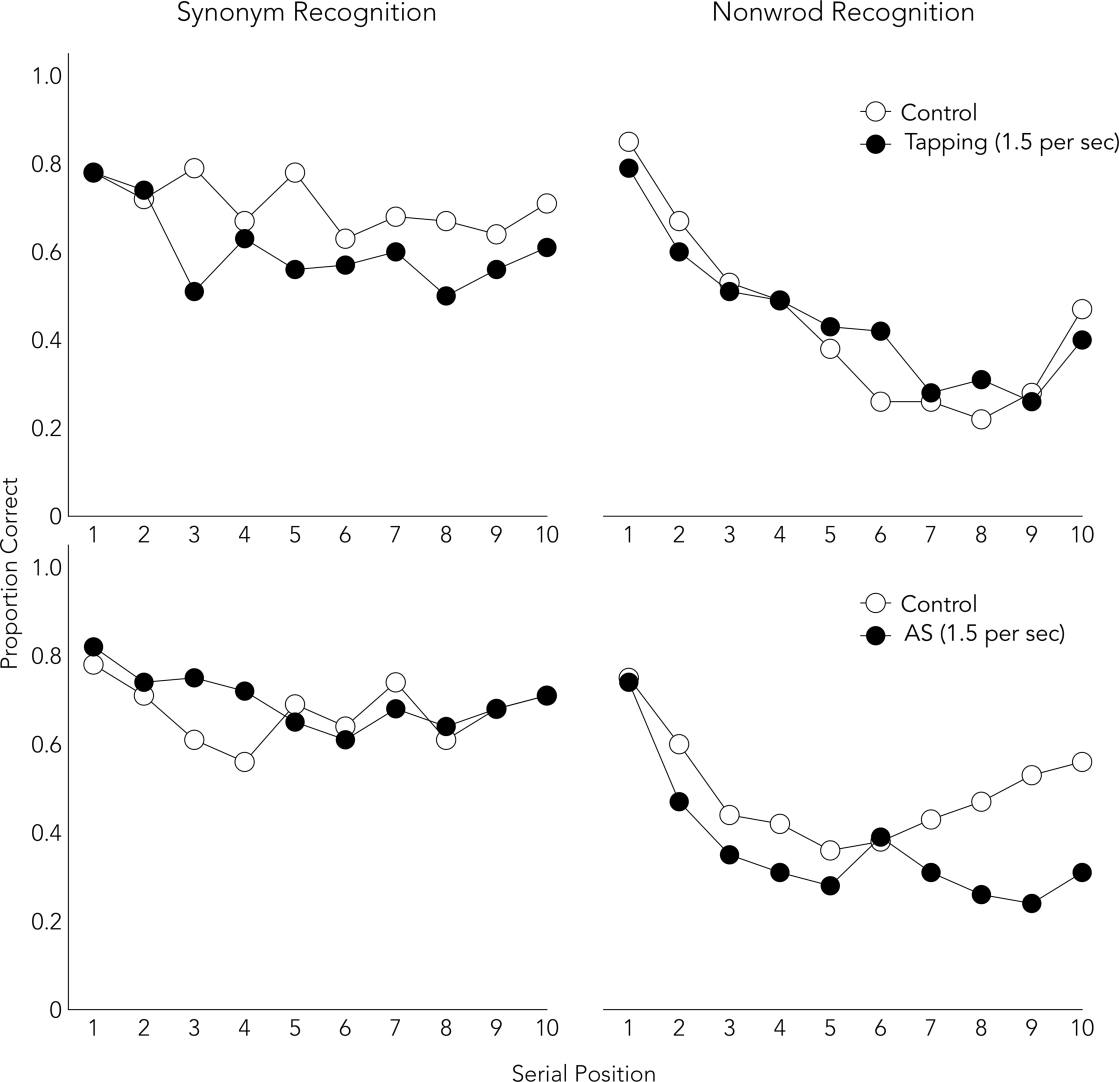
Serial position curves for both conditions in Experiments 2B.

Fig 3 shows the proportion of correct responses in each serial position. Tables 3 and 4 show the three-way interaction, which reflects selective disruption of tapping and AS on synonym and nonword recognition performance, respectively. This double dissociation is clear evidence of differences in the maintenance mechanisms for phonological and semantic representations of words.

## General discussion

First, tapping and AS disrupted synonym and nonword recognition performance respectively when these secondary tasks were performed during the study phase (Experiments 1A and 1C). This systematically replicated the double dissociation in the previous study [25] and suggests that different mechanisms are involved in active maintenance or encoding of semantic representations and phonological representations. Nevertheless, both secondary tasks disrupted both types of recognition performance when these were performed during the ISIs (Experiment 2A). This is not consistent with the results of Experiments 1A-1C because the interference effects of the secondary tasks increased even though the duration of the secondary tasks decreased. However, the double dissociation reappeared when the secondary tasks were performed during the delay before the recognition tests (Experiment 2B). Secondary tasks performed during the delay should compete not with encoding but only with maintenance; therefore, this confirms that the double dissociation occurs during maintenance activities. This supports the view that different mechanisms may handle the maintenance of semantic representations and of phonological representations. Nevertheless, there is a conflict between the results of Experiment 2A and Experiment 2B. I will discuss this conflict as well as the other results based on the characteristics of two active maintenance mechanisms in the next section.

### Active phonological maintenance

Traditional models of verbal WM assume that a loop of speech production and perception activities provides active phonological maintenance (e.g., articulatory loop: [12]). This seems to explain the significant disruption cause by AS (Experiments 1B, 1C, 2A, and 2B) and the null disruption caused by tapping (Experiments 1A and 2B) on nonword recognition performance as follows. Participants may rely on articulatory rehearsal to perform the nonword recognition task because such rehearsal is suitable for maintaining nonwords. This maintenance mechanism cannot adequately function concurrently with other articulatory activities (e.g., [16]). So, it is natural that AS disrupted nonword recognition performance. In contrast, while articulatory rehearsal is highly automatized and carried out without attentional control [14], tapping demands not articulatory activity but attentional control [28–30]. So, the models explain why tapping did not disrupt nonword recognition performance.

In addition, the phonological loop model also supposes that initiation of the speech production program demands some attentional control [47]. In other words, articulatory rehearsal can be impaired by additional attentional demands only at the initiation. This characteristic may account for the disruption of nonword recognition performance by tapping during ISIs (Experiment 2A). That is, tapping during ISIs might require control of finger movement to synchronize with the ISIs. If so, it might compete with the initiation of speech production programs for articulatory rehearsal. This interpretation is supported by the finding that syncopated tapping impaired the phonologically similarity effect, which is an index of phonological maintenance [28, 48, 49]. This interpretation is also consistent with the fact that AS during ISIs severely disrupted nonword recognition performance, because AS during ISIs can compete with both articulatory activities and the initiation of speech production programs.

### Active semantic maintenance

Current models of WM, i.e., *attentional refreshing* (e.g., [33, 34, 50]), can account for the other part of the present results, i.e., the significant disruption by tapping and the null disruption by AS of synonym recognition performance. The models assume that rethinking of an item in memory can refresh its representation, but concurrent activities which demand attentional resources, e.g., tapping, can compete with the attentional refreshing. However, attentional refreshing can be performed concurrently with automatized articulatory activities [34, 51]. The instructions for the synonym recognition task were to keep study words in mind by imaging (thinking of) their meanings. This aligns with the concept of attentional refreshing. In other words, attending to semantic representations can provide active semantic maintenance. This view is also consistent with WM models that assume attentional control sustains activated portions of LTM (e.g., [7, 32, 52]). Some evidence supports the relationship between attentional refreshing and active semantic maintenance. For instance, Haarmann, Davelaar, and Usher [6] reported a significant correlation between performance of semantic cued recall and reading span [53]. In the former task, active semantic maintenance assumedly plays an important role, and in the latter task attentional refreshing assumedly plays an important role. Also, Miyake, Just, and Carpenter [54] reported that individuals with high reading span scores were better at maintaining multiple meanings of a word.

Explanations for the present results based on the attentional refreshing account are as follows. Tapping demands some attentional control, which can decrease opportunities for repeatedly thinking about semantic representations. So, tapping disrupted synonym recognition performance. In contrast, automatized articulatory activities can be performed independently of other (non-phonological) cognitive activities [14]. So, AS did not disrupt synonym recognition performance. However, a fast rate of AS (Experiment 1B) or intermittent AS (Experiment 2) can demand some attentional control, and hence, disrupted synonym recognition performance.

Attentional refreshing is assumed to function independently from articulatory rehearsal [34], which is consistent with the present double dissociation. Nevertheless, semantic and phonological representations can interactively affect active maintenance of words. For instance, articulatory rehearsal of a word can give rise to its lexical-phonological representations, which evoke corresponding semantic representations; in turn, feedback from semantic representations then supports articulatory rehearsal [9, 52, 55].

### Alternative explanation

Performing the secondary tasks may evoke representations which correspond to the cognitive process, such as performing AS evoking phonological representations of “The.” Such representations might cause interference with to-be-maintained representations when these representations overlap. However, this cannot explain the disruption by tapping of synonym recognition performance because tapping does not evoke semantic representations; therefore, representations evoked through tapping cannot interfere with the to-be-maintained representations for synonym recognition. In sum, this explanation can account for only the half of the present results, the disruption by AS of phonological recognition performance.

Another alternative explanation is that the secondary tasks might compete with retrieval activities during the delay periods. The present study used 10 study words; hence, all words could not be concurrently kept in mind. So, some words might have been dropped from the conscious mind, and then attentional refreshing or articulatory activities could have brought them back. In other words, the present study has a limitation with regard to the ability to distinguish the secondary task effects on maintenance activities from their effects on retrieval activities. However, current models of working memory include such processes as memory maintenance activities (e.g., [56]).

Another limitation of this study is the relatively small sample size, even though *BF*s showed significant effects. Performing post-hoc power analyses may verify the results; however, this could be inappropriate (e.g., [57]). Rather, actual replications of the result would confirm its validity. In other words, Experiments 1A and 1C were replications of a previous study. So, it is likely certain that AS and tapping throughout the study phase can selectively disrupt semantic and phonological maintenance. Nevertheless, the selective disruptions of the secondary tasks during the delay phase should be replicated in subsequent studies.

Another limitation is that the only evidence suggesting that different memory mechanisms were used to perform the synonym and phonological recognition tasks is the interference effects of the secondary tasks. To provide additional support, serial position curves were displayed for each condition of each experiment (Figs 1–3). The synonym recognition task showed somewhat flat curves, whereas the phonological tasks showed U-shaped curves. The flat pattern is consistent with the previous report [40]. The U-shaped curve has been observed in verbal short-term memory studies which assume that participants use articulatory rehearsal [58, 59]. Moreover, the attenuation of a recency effect by the delay in Experiment 2B (Fig 3) replicated the previous finding in serial and free recall tasks [60, 61]. These findings support the view that different memory mechanisms were used to perform synonym and other phonological recognition tasks.

### Conclusion

The present study demonstrated selective impairment in performance of synonym recognition by tapping even though this secondary task cannot compete with semantic encoding. This is strong evidence that different maintenance mechanisms handle semantic and phonological representations when keeping words in mind. Attentional refreshing is the most likely candidate for the maintenance mechanism for semantic representations. In addition, all dual-task effects on active phonological maintenance observed in this study were within ranges consistent with the models of verbal WM, e.g., articulatory loop [12]. Therefore, the present findings can offer a basis for integrating findings regarding verbal working memory.

## Supporting information

S1 Table Recoded rates of secondary task. (DOCX)
