# Supplementary material for: Separability of active semantic and phonological maintenance in verbal working memory
Source: PLoS One. 2018 Mar 7;13(3):e0193808. doi: 10.1371/journal.pone.0193808 (PMC5841779; doi:10.1371/journal.pone.0193808)
Supplement: S1 Table — (DOCX) [file pone.0193808.s001.docx]

Recoded rates of secondary task

|  |  | Demanded rate: | Actual rate: per sec | |  |
| --- | --- | --- | --- | --- | --- |
|  | Secondary task | per sec | Synonym (*SD*) | Nonword (*SD*) | *t value* |
| Exp.1A | Tapping | 2 | 2.25 (0.16) | 2.34 (0.13) | 1.23 *ns* |
| Exp.1B | AS | 2 | 1.99 (0.18) | 1.99 (0.21) | 0.15 *ns* |
| Exp.1C | AS | 1 | 0.97 (0.08) | 0.96 (0.08) | 0.93 *ns* |
| Exp.2A | Tapping | 2 | 2.34 (0.09) | 2.43 (0.09) | 1.02 *ns* |
| Exp.2A | AS | 1 | 1.00 (> 0.01) | 1.00 (> 0.01) | 1.39 *ns* |
| Exp.2B | Tapping | 1.5 | 1.52 (0.01) | 1.51 (0.01) | 1.17 *ns* |
| Exp.2B | AS | 1.5 | 1.50 (0.01) | 1.51 (> 0.01) | 0.45 *ns* |
|  |  |  |  |  |  |

*Note*. AS = Articulatory suppression. Degrees of freedom were 11.
